# Wnt/β-catenin signaling pathway activation is required for proliferation of chicken primordial germ cells *in vitro*

**DOI:** 10.1038/srep34510

**Published:** 2016-09-30

**Authors:** Hyung Chul Lee, Sumi Lim, Jae Yong Han

**Affiliations:** 1 Department of Agricultural Biotechnology, College of Agriculture and Life Sciences, and Research Institute of Agriculture and Life Sciences, Seoul National University, Seoul 08826, Korea; 2 Department of Cell and Developmental Biology, University College London, London, United Kingdom; 3 Institute for Biomedical Sciences, Shinshu University, Minamiminowa, Nagano 399-4598, Japan

## Abstract

Here, we investigated the role of the Wnt/β-catenin signaling pathway in chicken primordial germ cells (PGCs) *in vitro*. We confirmed the expression of Wnt signaling pathway-related genes and the localization of β-catenin in the nucleus, revealing that this pathway is potentially activated in chicken PGCs. Then, using the single-cell pick-up assay, we examined the proliferative capacity of cultured PGCs in response to Wnt ligands, a β-catenin-mediated Wnt signaling activator (6-bromoindirubin-3′-oxime [BIO]) or inhibitor (JW74), in the presence or absence of basic fibroblast growth factor (bFGF). WNT1, WNT3A, and BIO promoted the proliferation of chicken PGCs similarly to bFGF, whereas JW74 inhibited this proliferation. Meanwhile, such treatments in combination with bFGF did not show a synergistic effect. bFGF treatment could not rescue PGC proliferation in the presence of JW74. In addition, we confirmed the translocation of β-catenin into the nucleus by the addition of bFGF after JW74 treatment. These results indicate that there is signaling crosstalk between FGF and Wnt, and that β-catenin acts on PGC proliferation downstream of bFGF. In conclusion, our study suggests that Wnt signaling enhances the proliferation of chicken PGCs via the stabilization of β-catenin and activation of its downstream genes.

Primordial germ cells (PGCs), the precursors of sperm or oocytes, undergo numerous spatially and temporally specific regulatory pathways during early development^1^. It has been revealed that these complex regulatory networks in PGCs are governed by several extrinsic factors leading to intracellular pathways^2^. Although the molecular mechanisms underlying signal transduction in PGCs remain poorly defined, leukemia inhibitory factor, basic fibroblast growth factor (bFGF), bone morphogenetic protein 4, and kit ligand are the best studied signaling pathways known to control PGC survival and proliferation^3^.

Previously, several studies reported a list of PGC specific genes by transcriptome analysis containing Wnt/β-catenin signaling-related genes^4^,^5^. Also, while we were searching for target genes regulated by micro RNA (miRNA) specifically expressed in PGCs based on the previous result^6^, we found that several negative regulators for Wnt signaling pathway, such as Glycogen synthase kinase-3-β (GSK3β), APC, and AXIN, might be target for those miRNAs, indicating possible role of Wnt pathway in chicken PGCs.

The Wnt pathway is a well-known signaling cascade that is involved in PGC development including specification, migration, and proliferation^7^,^8^,^9^,^10^. The Wnt/β-catenin pathway, or so-called canonical Wnt signaling, maintains embryonic stem (ES) cells in an undifferentiated state and promotes proliferative capacity in both stem cells and cancer cells^11^,^12^. β-catenin is a key mediator of canonical Wnt signaling, and acts as a transcriptional co-activator to control the expression of cell cycle-related genes^13^,^14^. The importance of Wnt/β-catenin signaling in the proliferation of PGCs has been reported in mice and *Drosophila*^8^,^15^,^16^, but not in chickens.

Several inhibitors, such as 6-bromoindirubin-3′-oxime (BIO) and JW74, have been utilized to activate or block such signaling. BIO, an inhibitor of the negative regulator GSK3β, leads to target gene expression by stabilizing β-catenin from the degradation complex^11^,^17^, whereas JW74 stabilizes AXIN, a component of the β-catenin degradation complex, eventually causing β-catenin degradation^18^. Accordingly, BIO and JW74 were used in this study to modulate the level of β-catenin in chicken PGCs *in vitro*.

Another growth factor family involved in cell proliferation, migration, and differentiation during embryonic development is bFGF, also known as FGF2^19^,^20^. bFGF is a powerful mitogen in PGC culture in mammals^21^ and supports the proliferation of embryonic germ cells^22^ and PGCs in chickens^23^. Choi *et al.*^24^ and Macdonald *et al.*^25^ identified bFGF as an essential factor for *in vitro* PGC proliferation^24^,^25^. Among several downstream cascades of bFGF, including the PI3K/AKT and MEK/ERK pathways, chicken PGCs mediate MEK/ERK signaling for proliferation^24^. Recently, Whyte *et al.*^26^ reported that three different signals, such as TGF beta, FGF, and Insulin are required for PGC proliferation in chicken, suggesting presence of combinatorial action of several signaling pathways^26^. Meanwhile, numerous recent studies have demonstrated the crosstalk between FGF and Wnt/β-catenin signaling. For example, β-catenin is activated by FGF signaling through the RAS/ERK or PI3K/AKT cascade, and acts downstream of these pathways in several stem cells or progenitor cells^27^,^28^,^29^,^30^. In addition, FGF can activate Wnt signaling by phosphorylation of the Wnt co-receptor LRP6^31^,^32^ or inactivation of the Wnt inhibitor Dkk1^33^.

In this study, the expression of Wnt/β-catenin signaling-related genes was examined to determine whether PGCs are capable of activating this signaling pathway. BIO and JW74 were applied to modulate Wnt/β-catenin signaling in cultured chicken PGCs. Subsequently, an *in vitro* proliferation assay was performed in the presence of a Wnt ligand, BIO, JW74, or bFGF to verify the signaling mechanism underlying PGC proliferation in chickens. Lastly, to verify the crosstalk between bFGF and Wnt/β-catenin signaling in PGC proliferation, Western blotting and immunocytochemistry were used to examine the translocation of β-catenin into the nucleus, which was stimulated by bFGF after JW74 treatment.

## Results

### Expression of Wnt signaling-related genes in chicken PGCs

**Figure 1:**
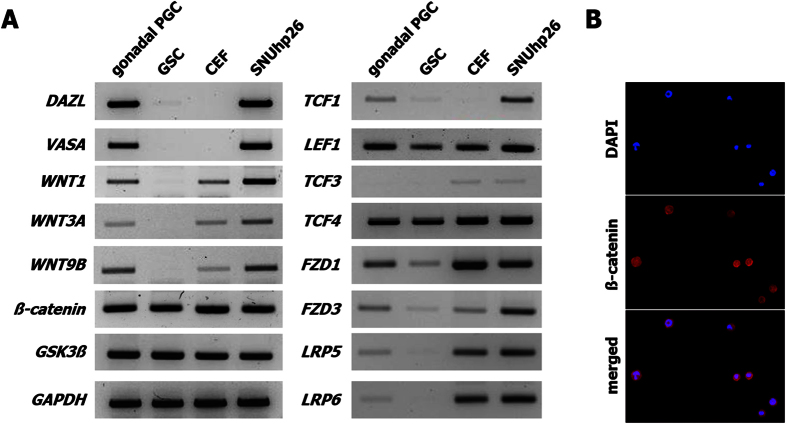
Expression of Wnt signaling pathway-related genes in cultured chicken primordial germ cells (PGCs). (**A**) The expression of Wnt signaling pathway-related genes including *WNT1*, *WNT3A*, *WNT9B*, *FZD1*, *FZD3*, *LRP5*, *LRP6*, *β-catenin*, *GSK3*β, *TCF1*, *LEF1*, *TCF3*, and *TCF4* and germ cell-related genes including *DAZL* and *VASA* was analyzed using reverse transcription polymerase chain reaction (RT-PCR) in gonadal PGCs and gonadal stromal cells (GSCs), which were separated from day 6 embryonic gonads, chicken embryonic fibroblasts (CEFs), and a cultured chicken PGC line, SNUhp26. Most of the Wnt signaling pathway-related genes were upregulated in SNUhp26 compared to those in gonadal PGCs. (**B**) Immunostaining showed the localization of β-catenin in both the nucleus and cytoplasm in cultured chicken PGCs.

To examine whether chicken PGCs have the ability to process Wnt/β-catenin signaling, RT-PCR was performed to analyze several components of this pathway. Among the many Wnt ligands and receptors, there was confirmed expression of WNT1, WNT3A, and WNT9B, which are well-known proteins in the canonical Wnt pathway; Frizzled receptors 1 and 3, which interact with WNT3A^34^,^35^; and LRP5 and 6, which are co-receptors of canonical Wnt signaling^36^,^37^,^38^ (Fig. 1A). In light of these findings, Wnt ligands and their receptors were well expressed in both gonadal and cultured PGCs (SNUhp26), and other key factors such as *β-catenin*, *GSK3*β, and *TCFs* (except for *TCF3*) were also expressed in gonadal stromal cells and chicken embryonic fibroblasts as well as gonadal PGCs and cultured PGCs, indicating that chicken PGCs might be capable of activating Wnt/β-catenin signaling (Fig. 1A). In particular, most Wnt signaling pathway-related genes, such as all three Wnt ligands, *FZDs*, *LRPs*, *TCFs*, and *LEF1*, were highly expressed in cultured PGCs compared to those in gonadal PGCs. To determine the status of β-catenin in PGCs, cultured PGCs were subjected to immunostaining (Fig. 1B). The results showed that β-catenin was localized in both the nucleus and cytoplasm, indicating that it is stabilized in cultured PGCs.

### Verification of the role of Wnt/β-catenin signaling in chicken PGCs via BIO and JW74

**Figure 2:**
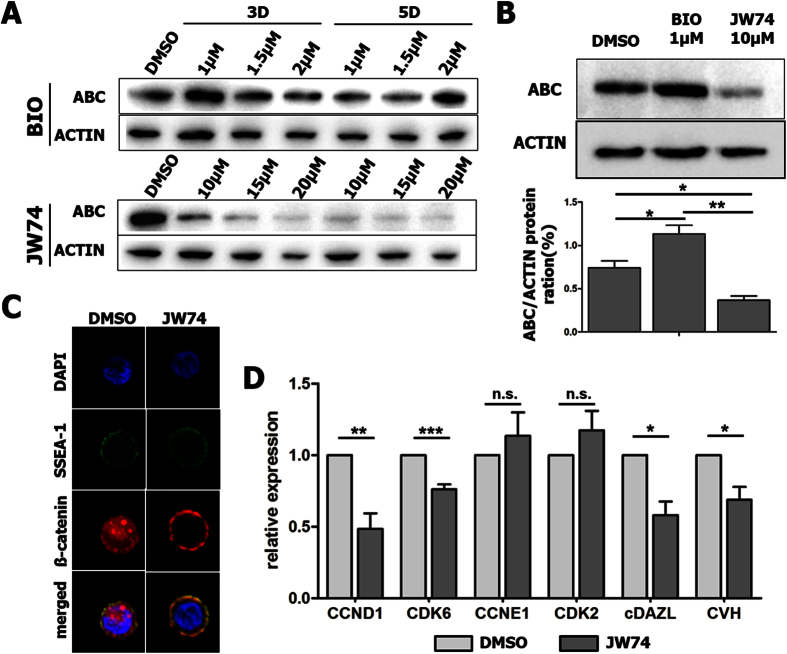
Regulation of β-catenin signaling by 6-bromoindirubin-3′-oxime (BIO) or JW74 treatment for optimization of chicken PGCs (SNUhp26) *in vitro*. (**A**) The level of active β-catenin (ABC) changed after BIO or JW74 treatment in a dose-dependent manner, as determined by Western blotting. (**B**) BIO applied at 1 μM and JW74 at 10 μM for 3 days showed optimal effects to increase or decrease the β-catenin level. BIO, a GSK3β inhibitor; JW74, a β-catenin inhibitor. (**C**) Immunostaining showed that JW74 treatment caused the degradation of β-catenin in both the nucleus and cytoplasm in chicken PGCs. (**D**) JW74 treatment caused downregulation of cell cycle-related genes essential for entry in G_1_ phase (*CCND1* and *CDK6*) and germ cell-specific genes (*cDAZL* and *CVH*), but no effect on cell cycle-related genes regulating progression from G_1_ to S phase (*CCNE1* and *CDK2*) as confirmed by qRT-PCR. Values in (**B**) and (**D**) represent mean ± standard error of the mean (SEM) of triplicate analyses. *P < 0.05, **P < 0.01, and ***P < 0.001. n.s.; not significant.

In several types of cells, such as cancer cells and ES cells, BIO and JW74 have been used for modulating β-catenin as an activator and an inhibitor, respectively^17^,^18^,^39^. To test whether β-catenin also works in cultured chicken PGCs, BIO and JW74 were administered at different concentrations for 3 and 5 days (Fig. 2A). Their effects were detected by Western blotting with the antibody for active β-catenin (ABC), which is the non-phosphorylated form (Fig. 2A). Among the various conditions, 1 μM BIO showed optimal effect of Wnt signaling activation in cultured chicken PGCs when it was applied for 3 rather than 5 days (Fig. 2B). In the case of JW74, the levels of ABC protein gradually decreased in a dose-dependent manner. However, the level of β-actin also decreased when JW74 was applied at a concentration greater than 10 μM, meaning that it was toxic to chicken PGCs (Fig. 2A). Thus, 1 μM BIO and 10 μM JW74 were used for subsequent experiments in this study (Fig. 2B).

To clarify the importance of Wnt/β-catenin signaling in PGCs, loss-of-function studies were conducted by treating cultured PGCs with JW74. JW74 treatment caused the degradation of β-catenin in both the nucleus and cytoplasm, indicating that Wnt signaling had been inactivated (Fig. 2C). JW74 treatment also caused the downregulation of cell cycle-related genes essential for entry in G_1_ phase, such as *CCND1* and *CDK6*, and germ cell-specific genes, such as *cDAZL* and *CVH*, while no effect on cell cycle-related genes regulating progression from G_1_ to S phase, such as *CCNE1* and *CDK2* (Fig. 2D).

### Proliferation assay of chicken PGCs with Wnt ligand, BIO or JW74

**Figure 3:**
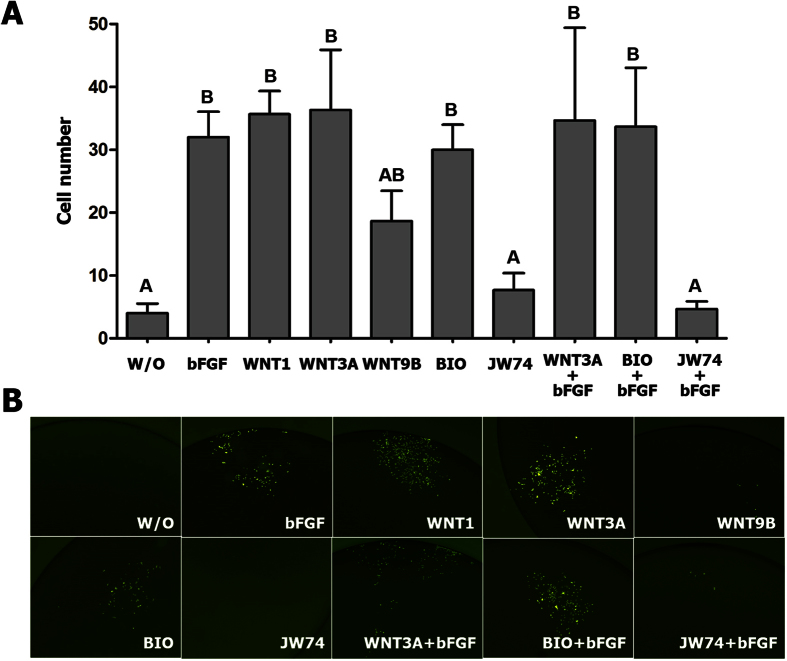
The effects of BIO and JW74 on the proliferation of cultured chicken PGCs (SNUhp26). Proliferation assay was performed with SNUhp26 PGCs, expressing green fluorescent protein (GFP). After bFGF starvation for 4 days, a single PGC was added to each medium containing bFGF, WNT1, WNT3A, WNT9B, BIO, or JW74 with or without bFGF, and cultured for 8 days. (**A**) The number of PGCs in each treatment was counted after 8 days. Values represent mean ± SEM of triplicate analyses. Values with different letters are significantly different (*P* < 0.05). (**B**) The GFP-expressing PGCs after each treatment were observed under a fluorescence microscope.

To investigate whether Wnt/β-catenin is involved in PGC proliferation *in vitro* and related to bFGF signaling in chicken PGCs, a Wnt ligand (WNT1, WNT3A, and WNT9B), BIO or JW74, were applied at different concentrations, after which a cell proliferation assay was performed (Fig. 3). To simplify determination of the cell number, SNUhp26 male chicken PGCs expressing GFP were used. After bFGF starvation for 4 days to remove all of the effects of bFGF signaling, a single PGC was added to a culture under each set of conditions. Then, the number of PGCs was counted on day 8 of culture, and GFP expression was observed (Fig. 3A,B). The results showed that, in the experimental sets with bFGF, WNT1, WNT3A, BIO, WNT3A with bFGF, and BIO with bFGF, cell number had significantly increased at day 8 compared to that in culture without a growth factor, in which GFP-expressing PGCs were finally lost after 10 days of culture. In the WNT9B group, an intermediate effect was shown. This indicates that β-catenin as well as bFGF can activate chicken PGCs to proliferate *in vitro* (Fig. 3A,B). On the other hand, JW74-treated PGCs did not grow, as the group without any growth factor showed the same pattern. In the group treated with JW74 supplemented with bFGF, this treatment could not rescue the inhibition of cell growth (Fig. 3A,B).

### Β-catenin-mediated activation of chicken PGCs through bFGF

**Figure 4:**
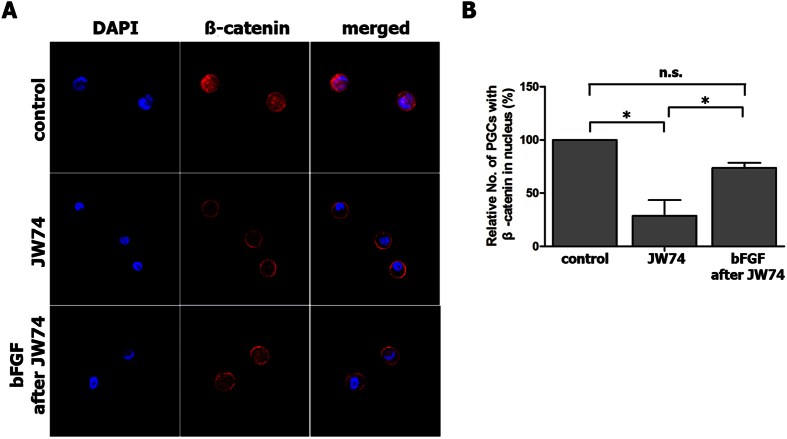
Activation and stabilization of β-catenin by bFGF treatment of chicken PGCs (SNUhp26). (**A**) Immunostaining for β-catenin showed that the JW74 treatment caused the degradation of β-catenin in both the nucleus and the cytoplasm in cultured chicken PGCs. When bFGF was added followed by JW74 treatment, β-catenin translocated into the nucleus from the membrane. (**B**) The relative number of PGCs with β-catenin located in the nucleus was counted after each treatment. *P < 0.05. n.s.; not significant.

Since bFGF is one of the key factors that enable the proliferation of chicken PGCs via MEK/ERK signaling^24^, we assumed that β-catenin is a downstream gene of bFGF in PGCs. To prove this, we used immunostaining to confirm the translocation of β-catenin into the nucleus from the cytoplasm (Fig. 4). After JW74 treatment (10 μM) for 2 days, most β-catenin in both the nucleus and cytoplasm had been degraded and was only detected in the membrane (Fig. 4A). When β-catenin had been almost completely cleared from the nucleus and cytoplasm, bFGF was added for 8 h. As a result, the translocation of β-catenin into the nucleus significantly increased (Fig. 4A,B), indicating that bFGF can activate and stabilize β-catenin to promote the proliferation of chicken PGCs.

## Discussion

Compared to other somatic cells, primordial germ cells have unique spatial and temporal developmental characteristics^1^, and several signaling pathways play important roles in regulating their development^2^. Among them, Wnt signaling has also been shown to be involved in PGC development in several model animals^16^,^40^,^41^, but not yet in chicken. In this study, based on the expression of Wnt signaling-related genes in chicken PGCs, we suggested that the Wnt pathway might play an important role in PGCs in chicken. Most Wnt signaling pathway-related genes were upregulated in cultured PGCs compared to those in gonadal PGCs, indicating that Wnt signaling might be more activated in these specific conditions, *in vitro*, to facilitate proliferation. In addition, β-catenin, an important mediator of Wnt signaling, was stabilized in cultured PGCs considering their localization in both the nucleus and the cytoplasm. These results suggest that chicken PGCs can activate and process the Wnt signaling pathway.

Aberrant activation of the canonical Wnt/β-catenin pathway is one of the most common signaling abnormalities in human and mouse cancer^42^,^43^, because it activates numerous genes related to cell cycle progression. To investigate the role of the Wnt/β-catenin pathway in chicken PGCs, we used BIO and JW74, well-known inhibitors of GSK3β and β-catenin, respectively^17^,^18^. We confirmed that both BIO and JW74, respectively, could activate and inactivate β-catenin in cultured PGCs by modulating the level of its active form (ABC). When β-catenin is activated and stabilized in the cytoplasm, it is transported into the nucleus, leading to formation of a transcriptional complex with the TCF transcription factor^44^. Specific gene expression mediated by β-catenin activation generally occurs in a cell-context and cell-dependent manner. Cyclin D1, MYC, and cJun are the best-known target genes that enhance proliferation^45^. In this study, to investigate the role of Wnt/β-catenin signaling, loss-of-function studies were conducted by applying JW74. The results suggest that inhibition of Wnt/β-catenin signaling induces cell cycle arrest by downregulation of CCND1 and CDK6^46^, revealing that Wnt/β-catenin signaling is required for the maintenance of PGC proliferation.

Next, to test whether Wnt/β-catenin is involved in the proliferation of chicken PGCs, we activated the Wnt signaling pathway in PGCs using Wnt ligands or the activation of a component downstream of it, β-catenin, using BIO. The results of our proliferation assay suggested that the activation of Wnt/β-catenin signaling induced the proliferation of PGCs *in vitro*, while JW74 treatment prevented this. Also, the proliferative ability induced by the activation of Wnt/β-catenin signaling was similar as those induced by bFGF, which is known as a major proliferation inducer in chicken PGCs^23^,^24^,^25^. These results suggest that Wnt/β-catenin signaling is required for PGC proliferation *in vitro*.

Our proliferation assay confirmed two other findings. First, no synergistic effect was observed upon the treatment of Wnt ligands or BIO in combination with bFGF, suggesting that bFGF and Wnt do not act independently of each other, and that PGCs already reach the threshold of proliferation capacity in the presence of either bFGF or Wnt alone. Second, bFGF treatment could not rescue PGC proliferation in the presence of JW74 (in the absence of Wnt/β-catenin signaling), indicating that bFGF helps PGCs to proliferate through direct activation of the downstream Wnt/β-catenin signaling pathway. Previous studies on the crosstalk between β-catenin and bFGF support our results. For example, in the absence of bFGF, β-catenin is only located in the membrane with E-cadherin, whereas it accumulates and is stably translocated into the nucleus to activate downstream genes in the presence of bFGF^14^,^27^. In the presence of bFGF, β-catenin helps neural stem cells proliferate when it is overexpressed, while enhancing neuronal differentiation in the absence of bFGF^28^. Moreover, bFGF increases β-catenin signaling in neural progenitor cells via multiple mechanisms: increasing the level of β-catenin mRNA, nuclear translocation of β-catenin, and phosphorylation of GSK-3β^47^. Because the role of β-catenin in the proliferation of chicken PGCs was recently reported^48^, in this study, we focused on evaluating the crosstalk between bFGF and β-catenin in chicken PGCs. First, we applied JW74 for the degradation of β-catenin and then added bFGF to the culture medium. Our results showed the reactivation of β-catenin by bFGF treatment after JW74. These results suggest that bFGF signaling promotes the proliferation of chicken PGCs via the activation of β-catenin and its downstream signaling. Many studies have shown that β-catenin is a target gene of FGF signaling through RAS/ERK or PI3K/AKT cascades^27^,^28^,^29^,^30^. Therefore, it is worthwhile studying which downstream signals are involved in their crosstalk in PGCs.

In our study, the role of the Wnt/β-catenin signaling pathway was investigated for the first time in chicken PGCs, and the relationship between Wnt/β-catenin and bFGF was revealed. We concluded that Wnt/β-catenin can enhance the proliferation of chicken PGCs, and act as a factor downstream of bFGF signaling. Interestingly, in mouse, Wnt/β-catenin signaling is suppressed and deleterious for PGC proliferation^8^. Thus, our results suggest that, although many key signaling pathways for PGC development are conserved among animal species, the specific mechanism of regulation could differ. In addition, the recent report suggests the requirement of combinatorial action of different signals on PGC self-renewal^26^. Thus, a following study could be investigation on epistasis and crosstalk among different signaling pathways, such as TGF beta, FGF, Insulin, and WNT on PGC proliferation. Taken together, this research should contribute to studies on PGC biology and provide a comprehensive understanding of the mechanism behind PGC development from an evo-devo perspective.

## Methods

### Experimental animals and animal care

The care and experimental use of chickens were approved by the Institute of Laboratory Animal Resources, Seoul National University (SNU-150827-1). The chickens were maintained in accordance with a standard management program at the University Animal Farm, Seoul National University, South Korea. The procedures for animal management, reproduction, and embryo manipulation adhered to the standard operating protocols of our laboratory.

### PGC isolation and culture

To conduct gene expression analysis, PGCs from White Leghorn embryonic gonads at day 6 (stage 28) were isolated by magnetic activated cell sorting as previously reported^49^. Anti-stage-specific embryonic antigen (SSEA)-1 antibody-positive cells were regarded as gonadal PGCs, while anti-SSEA-1 antibody-negative cells were regarded as gonadal stromal cells (GSCs). For *in vitro* culture, SNUhp26 male line cultured PGCs^50^, expressing green fluorescent protein (GFP), were maintained with knockout Dulbecco’s Modified Eagle Medium (Thermo Fisher–Invitrogen, Carlsbad, CA, USA) supplemented with 20% (vol/vol) fetal bovine serum (Invitrogen), 2% (vol/vol) chicken serum (Sigma-Aldrich, St. Louis, MO, USA), 1× nucleosides (Millipore, Temecula, CA, USA), 2 mM L-glutamine, 1× nonessential amino acids, β-mercaptoethanol, 10 mM sodium pyruvate, and 1× antibiotic–antimycotic (Invitrogen). Human bFGF (Koma Biotech, Seoul, South Korea) was used at 10 ng/mL for PGC self-renewal. Chicken PGCs were cultured in an incubator maintained at 37 °C with an atmosphere of 5% CO_2_ and 60–70% relative humidity. The cultured PGCs were subcultured onto mitomycin-inactivated murine embryonic fibroblasts in 5 to 6-day intervals by gentle pipetting without any enzyme treatment.

### RT-PCR and qRT-PCR analyses

Total RNA was extracted from each sample with Trizol reagent (Invitrogen), in accordance with the manufacturer’s protocol. The RNA quantity was determined by spectrophotometry at 260 nm, and 1 μg of each RNA sample was reverse-transcribed with the Superscript III First-Strand Synthesis System (Invitrogen). The cDNA was diluted five-fold and quantitatively equalized for PCR amplification. RT-PCR was conducted to examine the change in expression of candidate genes in cultured chicken PGCs using specific primer sets (Table 1). PCR amplification was performed as follows: 94 °C for 5 min, followed by 26–32 cycles at 94 °C for 30 s, 58–62 °C for 30 s, and 72 °C for 30 s. The PCR products were mixed with SYBR^®^ Safe (Invitrogen) and loaded for gel electrophoresis. Confirmation of the results was performed using the ChemiDoc XRS System (Bio-Rad Laboratories, Hercules, CA, USA). Downregulation of cell cycle- or germ cell-related genes at the mRNA level was analyzed using quantitative RT-PCR (qRT-PCR). Relative expression was calculated after the threshold cycle had been normalized to that of chicken glyceraldehyde 3-phosphate dehydrogenase.

### Western blot analysis

Total protein was extracted with the CytoBuster^TM^ Protein Extraction Reagent (Novagen, Darmstadt, Germany), separated on a 10% polyacrylamide gel, and transferred to a polyvinylidene fluoride membrane (Millipore). The primary antibodies were mouse anti-β-actin (Santa Cruz Biotechnology, Santa Cruz, CA, USA) and rabbit anti-non-phospho (Active) β-catenin (Ser33/37.Thr41) (Cell Signaling, Danvers, MA, USA). Horseradish peroxidase-conjugated anti-mouse IgG and anti-rabbit IgG (Santa Cruz) were used as the secondary antibodies. The blots were treated with the enhanced chemiluminescence substrate solutions and exposed to a ChemiDoc XRS System (Bio-Rad) to detect chemiluminescence.

### Proliferation assay by single-cell pick-up

SNUhp26 male line cultured PGCs, expressing GFP, were used for evaluating the rate of proliferation^50^. The SNUhp26 has a full germ cell characteristics and potency that can produce germline chimera and its progeny^50^. A total of 10 culture conditions were prepared in 96-well plates as follows: no growth factor (w/o GF), bFGF (10 ng/mL), WNT1 (100 ng/mL; Abcam, Cambridge, UK), WNT3A (100 ng/mL; Cell Signaling), WNT9B (400 ng/mL; R&D Systems, Minneapolis, MN, USA), BIO (Sigma; B1686), JW74 (Sigma; SML0227), WNT3A with bFGF, BIO with bFGF, and JW74 with bFGF. After 4 days of bFGF starvation, a single PGC was picked up and added to culture under each of the above conditions. The final concentrations of BIO and JW74 were 1 and 10 μM, respectively. After 8 days of culture, the number of PGCs was counted by fluorescence microscopy.

### Immunocytochemistry

Cells were moved from the culture plate and dried on HistoBond^®^ adhesive microscope slides (Marienfeld-Superior, Lauda-Königshofen, Germany) for approximately 30 min. Then they were fixed in 100% methanol and prechilled for 3 min at −20 °C. After washing the cells three times for 10 min each in phosphate-buffered saline containing 1% normal goat serum, they were incubated in diluted anti-β-catenin antibody (mouse IgG) or anti-SSEA-1 (mouse IgM) antibody as a primary antibody at 4 °C for 24 h, and then incubated again in diluted Alexa-IgG-568 antibody or fluorescein isothiocyanate (FITC; Santa Cruz)-conjugated goat anti-mouse IgM, respectively, as a secondary antibody for 1 h at room temperature. Samples were then mounted with Prolong Gold antifade reagent with 4′,6-diamidino-2-phenylindole (Invitrogen) and visualized using fluorescence microscopy.

### Statistical analysis

Statistical analysis was performed using SAS version 9.4 software (SAS Institute, Cary, NC, USA). When a significant main effect was detected by analysis of variance using SAS, the least-squares analysis was used to compare the different treatments. Differences between control and treatment groups were deemed to be significant when *P* < 0.05.

## Additional Information

**How to cite this article**: Lee, H. C. *et al.* Wnt/β-catenin signaling pathway activation is required for proliferation of chicken primordial germ cells *in vitro. Sci. Rep.* **6**, 34510; doi: 10.1038/srep34510 (2016).

## Figures and Tables

**Table 1:** Primers used for RT-PCR.

Gene name	Description	Accession No.	Primer Sequences (5′ → 3′)	
			Forward	Reverse
*cDAZL*	deleted in azoospermia ― like	NM_204218.1	CTCCTGCACCGCAATTCCAT	TCTTTGCTCCCCAGGAACCC
*cVASA*	DEAD (Asp ― Glu ― Ala ― Asp) box polypeptide 4	NM_204708.1	CCGCATGCTCGATATGGGTT	TCCCTCTTGGATAACCGGGG
*WNT1*	wingless ― type MMTV integration site family, member 1	XM_015273182.1	TCTCAGGGCCCCAACATCTT	CGAAGAGCCTCCCGAAATCG
*WNT3A*	wingless ― type MMTV integration site family, member 3A	NM_001171601.1	AGGCCCTTTGTGGTGGGAGT	GAGGGGGTGAAGTTGGGCAT
*WNT9B*	wingless ― type MMTV integration site family, member 9B	XM_015299263.1	GCTTTCGCCTTTCCACGAGA	CACGTTCTCCCAGCAGTTCC
*FZD1*	frizzled family receptor 1	NM_001030337.1	CTGCTTCGTGGGCATCAACA	AATGACGATGGTGGCCGGTA
*FZD3*	frizzled family receptor 3	NM_001271940.1	GGCTTTTTGTCACCCCTCCC	AATGTGCCCAGGTCACCCAG
*LRP5*	low density lipoprotein receptor ― related protein 5	NM_001012897.1	GCAAGAGCGAGCTCCCAAGA	AGGCCCATTGGCTGAAGGAT
*LRP6*	low density lipoprotein receptor ― related protein 6	XM_417286.4	CTGTATGCAAACCGCCGTGA	ATGCCAGCCCATCAGGTGAC
*ß ― catenin*	catenin (cadherin ― associated protein), beta 1, 88k	NM_205081.1	ATTTGTGCGCTCCGTCACCT	ACCTTGTTCACGCAGTGGGG
*GSK3ß*	glycogen synthase kinase 3	XM_416557.4	TCCATTCCTTTGGGATCTGCC	TACACAGCCCGCTGACCACA
*TCF1 (TCF7)*	transcription factor 7 (T ― cell specific, HMG ― box)	NM_204547.1	ATGAAAGAGATGCGGGCGAA	CTGCTGCTTTTCCCGTGTCC
*LEF1*	lymphoid enhancer ― binding factor 1	NM_205013.2	GCGGCTTGTACAGCAAGGGA	GGAGCCTGGGGAAAAGTGCT
*TCF3 (TCF7L1)*	transcription factor 7 ― like 1 (T ― cell specific, HMG ― box)	XM_003643906.2	GGAATGCTGATGTGGGACCG	CCCAAACTGTGGGACCGAAA
*TCF4 (TCF7L2)*	transcription factor 7 ― like 2 (T ― cell specific, HMG ― box)	NM_001206510.2	AAATCCCCCATCCGCTAGGA	AGCCGACGTCACTCTGGGAA
*CDK6*	cyclin-dependent kinase 6	NM_001007892.2	TGGGGCTGCTTCACATTGAC	GCCAGCAGCAAAACGTTCAG
*CCND1*	cyclin D1	NM_205381.1	GGTGCTGCAGACCATGCTGA	GCAATTGCAACCGGCTTTTC
*CDK2*	cyclin-dependent kinase 2	NM_001199857.1	GAGCTACCTGTTCCAGCTGC	CCAGATGTCCACAGCAGTCG
*CCNE1*	cyclin E1	NM_001031358.1	TCGAGTACGCCAGCCACTTA	GAAATGTCGCCTTGCACAGC
*GAPDH*	glyceraldehyde ― 3 ― phosphate dehydrogenase	NM_204305.1	GGTGGTGCTAAGCGTGTTAT	ACCTCTGTCATCTCTCCACA
